# Machine learning-enabled inverse design of bioinspired layered composite structures with maximum auxetic performance

**DOI:** 10.1038/s44172-025-00557-5

**Published:** 2025-11-24

**Authors:** Yuze Li, Rui Li, Yin Fan, Zhouyu Zheng, Hui-Shen Shen, Xiuhua Chen, Minhua Wen, James Lin, Woong-Ryeol Yu, Yeqing Wang

**Affiliations:** 1 School of Aeronautics and Astronautics, Shanghai Jiao Tong University, Shanghai, China; 2 High Performance Computing Center, Shanghai Jiao Tong University, Shanghai, China; 3 Department of Materials Science and Engineering, Research Institute of Advanced Materials, Seoul National University, Seoul, Republic of Korea; 4 Department of Mechanical and Aerospace Engineering, Syracuse University, Syracuse, NY USA

**Keywords:** Mechanical engineering, Composites, Computational science, Bioinspired materials

## Abstract

Layered composite structures inspired by biological tissues can exhibit out-of-plane negative Poisson’s ratio, but identifying layups that maximize auxetic performance is challenging in high-dimensional designs. Here, we introduce an inverse design framework that searches for laminate layups with minimum Poisson’s ratio. The approach combines multi-start resampling with machine learning–guided clustering to map layup families across layer numbers. Analytical relations from laminate mechanics link ply angles to effective properties, and computer simulations with laboratory measurements validate the predicted minima. The analysis resolves three layup categories, explains how shear-strain mismatch across bonded plies drives through-thickness auxetic expansion, and shows that simple symmetry rules reduce the search space. The framework reproduces previously reported minima and uncovers layups that approach lower Poisson’s ratios under practical constraints. These results provide a physics-grounded, data-efficient route to engineer layered composite structures with strong auxetic responses and offer concise design rules for impact mitigation, vibration control, and flexible structures.

## Abstract (web summary)

Identifying layup configurations that achieve maximum auxetic characteristics and thereby maximize performance enhancements is challenging. Yuze Li and colleagues introduce an intelligent machine learning–aided inverse design method to efficiently identify layups with optimal auxetic performance.

## Introduction

**Fig. 1:**
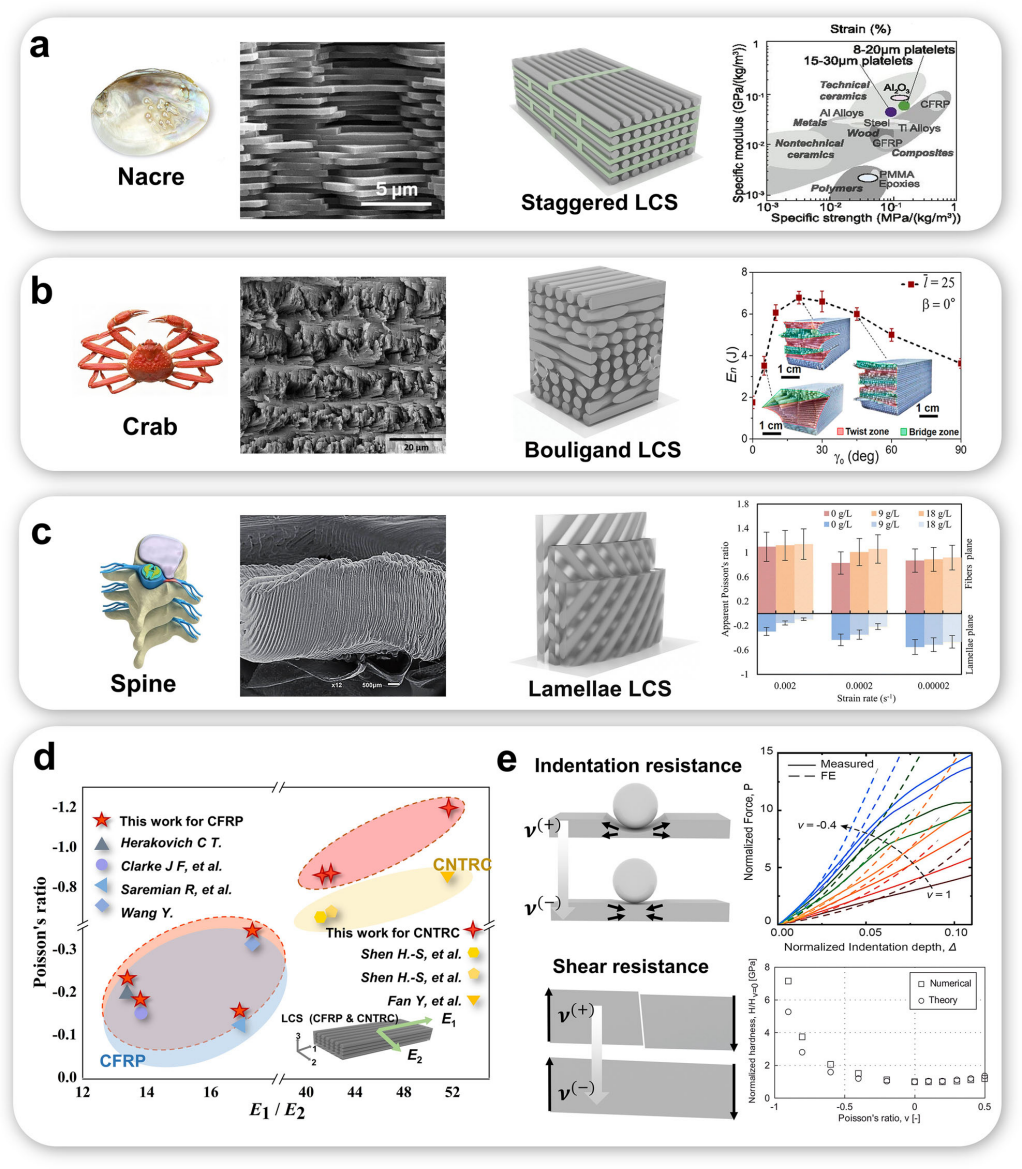
Mechanical optimization of biomimetic layered composite structures (LCSs) inspired by natural biostructures. Layered biostructures existing in nature found in (**a**) nacre, (**b**) crab and (**c**) spine. Each panel includes Specific organisms, Scanning Electron Microscope image of the natural materials’ microstructure^1–3^, the infill pattern model drawing, and their corresponding biomimetic LCSs with various excellent mechanical properties. In particular, Staggered LCSs^4^ can be made more than $20 \%$ higher than previously reported for brick-and-mortar polymer matrix composites by incorporating the proportion of platelet-connected mineral bridges $(\gamma )$, and their modulus of elasticity, strength, and fracture toughness all increase with the fraction of mineral bridges; Bouligand LCSs^5^ are found that initial crack orientation $(\beta )$ and the pitch angle $\left({\gamma }_{0}\right)$ at specific values can improve energy dissipations (${E}_{n}$) with crack orientation insensitivity of the composites to obtain excellent fracture resistance; Lamellae LCSs^8^ exhibit out-of-plane auxetic due to the special fiber orientation thus highlighting the osmo-inelastic coupling. **d** Comparison of calculated negative Poisson’s ratio (NPR) in this work with the existing literature^19,21,22,28–30^. **e** Superiority of extreme auxetic behaviors in terms of indentation resistance^35^ and shear resistance^36^. (Images in panels (a, b, c and e) and related schematics or data are reproduced or adapted from refs. ^1–5,8,35,36^. with permission from Elsevier, Springer Nature, Wiley, IOP Publishing, and the National Academy of Sciences (USA). All rights reserved.

Layered structures, architected by nature and exhibiting strong mechanical performance, are found in biosomes such as nacre^1^, crab shells^2^, and the intervertebral discs of the spine^3^ (as depicted in Fig. 1a, b, c). Recently, these bioinspired concepts have been learned and introduced into conventional layered composite structures (LCSs) with arrangements of brick-and-motor (staggered structures)^4^, Bouligand^5–7^, and lamellae^8,9^ (as illustrated in Fig. 1a–c), respectively, to further improve their design and optimization space oriented to desired mechanical properties. Although learning design philosophy from nature is always an efficient and inspirational approach for humans, this also exposes the disadvantages and limitations of conventional design philosophy. Additionally, achieving optimal physical properties remains a challenge even if a biomimetic design concept is adapted for LCSs.

As shown in Fig. 1c, cross-ply lamellae of annulus fibrosus in intervertebral discs of the spine exhibiting negative Poisson’s ratio (NPR, also known as auxetic) effect^10–13^ through thickness direction that is essential load absorption and transmission for discs. Poisson’s ratio (PR), describing deformation response of materials or structures to external force is a paramount mechanical parameter in solid mechanics. The corresponding biomimetic LCSs with lamellae arrangement is able to possess out-of-plane NPR^8,14,15^ with a special way of stacking consequence in the thickness direction. Recent studies^16,17^ demonstrated that auxetic structures with out-of-plane NPR exhibit enhanced energy absorption. This property is crucial for applications requiring impact resistance and damping, making these structures highly valuable for advanced engineering applications. Previous studies^18–21^ have demonstrated that the special stacking sequence for achieving NPR can be theoretically determined under certain constraints (such as symmetry, antisymmetry, cross-ply et al.). This implies that the minimum NPR (i.e. maximum auxetic characteristic) can only be identified within a narrow scope rather than a global one, which is not advantageous for leveraging the optimal properties of LCSs.

Based on generalized Hooke’s law (GHL), the mathematical relationship between PR and lamination angle of layers in LCSs is constructed^21^. In conventional design idea, lamination angle for each layer is an input variable between −90° and 90° representing one dimension and all selected layups are calculated for the minimum NPR. However, when the dimension is high (i.e. more layers), it is impossible to solve the global minimum NPR through the idea. Therefore, we always try to find largest value among some given layups^22^. Obviously, the found value is local minimum as the stacking sequences is constrained in advance to strictly limit input number of variables. However, predicting the extreme value of the out-of-plane PR using this procedure is particularly challenging, especially when the number of layers is large, for example, aircraft composite structures frequently require an LCS of more than 16 layers. Paradoxically, the extremity that a physical parameter can achieve is always desired to fully explore material potential. Whereas the conventional design is not effective when the minimum NPR is unknown as well as the corresponding layup. In contrast, the inverse design strategy^23–27^ beginning with desired performance parameters and working backwards to deduce the material structures and layup configurations required-offers a fresh perspective on the design and application of composite materials. This method not only optimizes the design process but also highlights the importance of inverse design in tackling complex engineering challenges. It opens an effective pathway for precisely controlling material properties to meet specific application demands.

Owing to the complexity of the mathematical model for GHL, directly finding extreme values via partial derivatives in a high-dimensional space also seems impractical. This paper proposes a data-driven approach that combines a modified algorithm with machine learning (ML) to address the troubling design problem to engineers^25^: determining the minimum NPR and the associated layup for LCSs. More importantly, by assistance of the approach, the cluster of various layups for minimum NPR according to characteristics of stacking sequences reveals the physics behind it. Additionally, the model in this paper is capable of calculating the extreme values reported in previous literatures^19,21,22,28–30^. By plotting PR on the vertical axis and ${E}_{1}/{E}_{2}$ on the horizontal axis, the extremes for both carbon fiber reinforced polymer (CFRP) and carbon nanotube reinforced composite (CNTRC) types of LCS are computed, as shown in the Fig. 1d. The results indicate that the ${E}_{1}/{E}_{2}$ values for carbon nanotube reinforced composite are generally higher than those for carbon fiber reinforced polymer, and the NPR values calculated in this work exceed those reported in previous studies.

This work is expected to inspire more auxetically architectured thermosetting materials to be designed in the future for various applications, such as helmet liners^31,32^, soft robotics^10,33^, flexible electronics^34^ and so on. In particular, with increasing auxetic performance, the enhancement in the indentation resistance^35^ and shear resistance^36^ of the material becomes larger (as shown in Fig. 1e). Hence, designing materials to approach their ultimate auxetic potential holds particular significance. The exceptional indentation resistance stems from auxetic materials’ negative Poisson’s ratio-induced lateral expansion under compression, which effectively redistributes concentrated stresses and prevents localized deformation. This characteristic shows promising potential for developing next-generation protective equipment, including impact-absorbing automotive components and adaptive body armor systems that require both flexibility and penetration resistance. Meanwhile, the improved mechanical failure resistance, manifested through enhanced crack propagation resistance and fatigue durability, positions auxetic architectures as ideal candidates for flexible electronics substrates that demand long-term structural integrity under cyclic bending, as well as load-bearing biomedical implants requiring sustained mechanical performance. Emerging applications could extend to vibration-damping aerospace components where auxetic metamaterials’ energy dissipation capacity synergizes with their damage tolerance, and smart infrastructure systems that utilize their strain-responsive pore geometry for real-time pressure sensing. Future research directions may focus on optimizing multiscale structural hierarchies to amplify these synergistic benefits while maintaining material functionality under extreme deformation conditions.

## Methods

### Inverse design

**Fig. 2:**
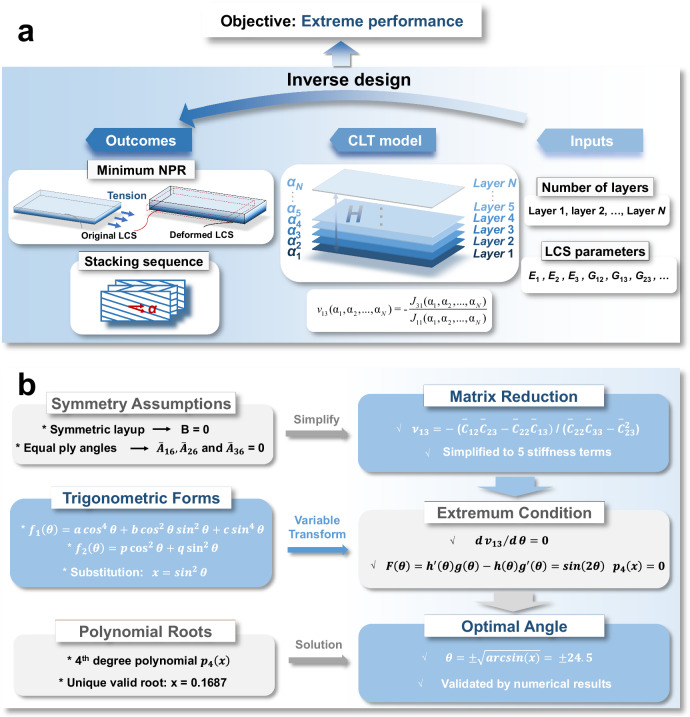
Schematic diagram of inverse design and mathematic proof process. **a** Inverse design for minimum negative Poisson’s ratio (NPR) of layered composite structure (LCS). **b** Mathematic proof process for inverse design results.

Regarding an LCS model depicted schematically in Fig. 2 consists of $N$ layers with total thickness $H$. The lamination angles for each layer, ${a}_{1},{a}_{2},\ldots ,{a}_{N}$, are unknown and needed to be determined for minimum out-of-plane NPR. Starting from basis of GHL, ${{{\bf{A}}}},{{{\bf{B}}}}$, and ${{{\bf{D}}}}$ stiffness matrices are well-known reflecting structural performance in stretch, stretch-bending, and bending, respectively. Their elements that are related to layer properties $p$ (details can be found in Table 1) and lamination angle $a$ are expressed mathematically as

1 $$\left({A}_{{ij}},{B}_{{ij}},{D}_{{ij}}\right)={\sum }_{k=1}^{N}\int _{-H/2}^{H/2}{C}_{{ij}}^{k}\left({p}_{k},{a}_{k}\right)\left({Z}^{0},{Z}^{1},{Z}^{2}\right){dZ},$$

$$\left(i,j=1,2,3,4;{-90}^{\circ }\le {a}_{k} < {90}^{\circ }\right)$$

where ${C}_{{ij}}^{k}$ are elastic stiffness coefficients of the *k*th layer (detailed expressions can be found in Supplementary Information, Discussion S1). Generally, the layer properties in an LCS are identical and given, thus the values of ${C}_{{ij}}$ are only determined by lamination angle. Then a $4\times 4$ ${{{\bf{J}}}}$ matrix (derivation can be found in Supplementary Information, Discussion S1) containing out-of-plane effect can be obtained from GHL based on foundation of strain-stress relationship

2 $${{{\bf{J}}}}={{{{\bf{A}}}}}^{-1}+{{{{\bf{A}}}}}^{-1}{{{\bf{B}}}}\left({{{\bf{D}}}}-{{{\bf{B}}}}{{{{\bf{A}}}}}^{-1}{{{\bf{B}}}}\right){{{\bf{B}}}}{{{{\bf{A}}}}}^{-1}.$$

**Table 1:** Material properties of IM7/977-3 unidirectional lamina

Thickness	${E}_{1}$	${E}_{2}$	${E}_{3}$	${\nu }_{12}$	${\nu }_{13}$	${\nu }_{23}$	${G}_{12}$
0.08 mm	159 GPa	9.2 GPa	9.2 GPa	0.253	0.253	0.45	4.37 GPa

At last, one of the out-of-plane PRs we concern can be expressed by the two elements (detailed expressions can be found in Supplementary Information, Discussion S1) in ${{{\bf{J}}}}$ matrix

3 $${\nu }_{13}\left({a}_{1},{a}_{2},\ldots ,{a}_{N}\right)=-\frac{{J}_{31}\left({a}_{1},{a}_{2},\ldots ,{a}_{N}\right)}{{J}_{11}\left({a}_{1},{a}_{2},\ldots ,{a}_{N}\right)}.$$

From the view of mathematics, Eq. 3 can be seen as a graph of the function with $N$ variables in a $(N+1)$ dimensional space. To find the minimum value of ${\nu }_{13}$, conventional design thought requires comparison of all extreme points in that high-dimension space. Obviously, solving complexity grows exponentially as number of layers increases if no constraints are applied. For this reason, most of studies about NPR of LCSs are implemented with constraints (i.e. symmetric, antisymmetric) on the stacking sequence.

Notably, physics-informed neural networks and neural networks-accelerated solvers are valuable in scenarios involving incomplete or partially known physical models^23,26,27^, their efficacy diminishes when applied to inverse problems characterized by explicit mathematical relationships. Specifically, the primary function of neural networks resides in establishing nonlinear mappings between input-output correlations through data-driven approximations. However, when problems are mathematically tractable, the computational expenditure required for generating extensive training datasets and optimizing network architectures becomes counterproductive from an efficiency standpoint. If one attempts to construct an inverse neural network to map equivalent Poisson’s ratios to laminate angles, the inherently ill-posed one-to-many mapping problem would arise, resulting in poor convergence, high model complexity, and physically inconsistent predictions. Therefore, neural network approaches cannot guarantee global convergence.

Thus, this study introduces a data-driven method to address the complexities in the design of LCSs by employing inverse design. Conventional design approaches typically initiate from problem identification and proceed linearly toward a solution. In contrast, inverse design^25,37^ begins with the desired solution and works backward to uncover the underlying causes. While methods based on GHL can determine the out-of-plane NPR from ply configurations, their limitations in managing complex, high-dimensional models hinder the prediction of extreme NPR values, which are essential for maximizing material performance, for instance, same absolute value of angles for each layer, fixed angle order.

Confronting this gap, our research leverages ML to inverse design process. Instead of starting with a predefined layup and exploring achievable performance, we begin with the goal of searching for the extreme value of NPR and employ algorithmic power to deduce the optimal layup configurations that can realize these targets. This inverse design, illustrated in Fig. 2a, represents a transformative shift in material design philosophy, where the end performance goal dictates the structural configuration, rather than the conventional approach of structure-led performance outcomes.

The inverse design results are subjected to rigorous mathematical validation to ensure their accuracy. The primary proof logic, shown in Fig. 2b, reveals that the optimized results are completely consistent with the inverse design outcomes, thereby verifying the reliability of our ML-based inverse design framework. For comprehensive details, please see Section S2 in the Supplementary Information.

### Multi-start resampling algorithm

**Fig. 3:**
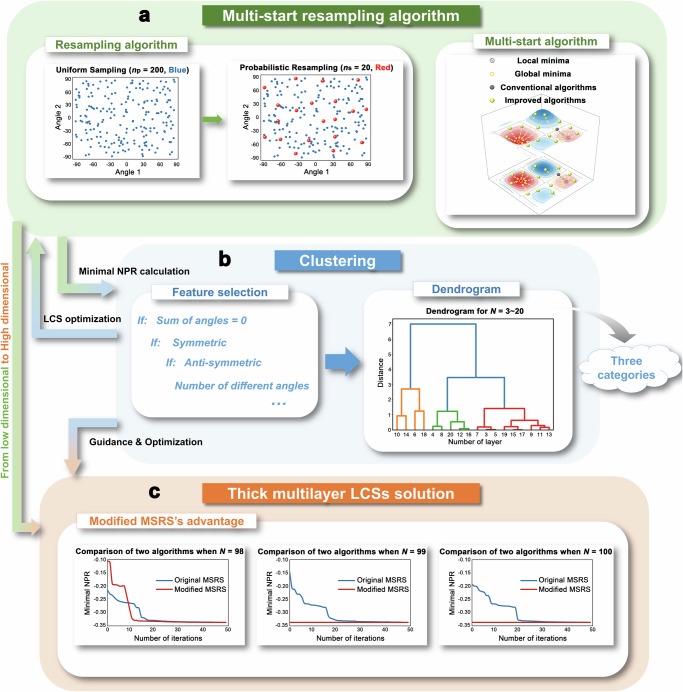
Deriving minimum negative Poisson’s ratio (NPR) in layered composite structure (LCS) configurations through machine learning (ML) approach Schematic of the ML method, including (**a**) the multi-start resampling algorithm (blue dots represent the initially generated uniform samples, and red dots denote the points obtained after probabilistic resampling in the multi-start algorithm) and (**b**) the clustering technique. **c** Improvement in computational efficiency for high-dimensional problems using the layup characteristics of these three categories.

The optimal solution to the problem mentioned above is not unique in mathematics; therefore, all possible solutions need to be identified. To address this, a multi-start resampling algorithm is proposed to search for the minimum NPRs and their corresponding layups in $N$-dimensional space (Fig. 3a). By initiating the process with multiple points in the solution domain and executing numerous iterations, this algorithm not only efficiently identifies the global optimal value but also reveals all optimal points. To mitigate the exponential complexity growth encountered with high-dimensional variables, we implement a resampling strategy on the initial points, thereby enhancing iteration efficiency. Additionally, leveraging the k-means++^38^ ML technique, the probability distribution of resampled points is adjusted to reflect distances from existing sampled points, ensuring a more representative distribution across the entire domain.

4 $$P\left({x}_{i}\right)=\frac{{D}^{2}\left({x}_{i}\right)}{{\sum}_{x\in \Omega }{D}^{2}(x)},$$

where $\Omega$ is sample space, *D*(*x*_*i*_) is the minimal distance from *x*_*i*_ to all sampled points. The resampled vectors are chosen as initial points, and iterations are subsequently conducted using the Broyden–Fletcher–Goldfarb–Shanno algorithm^39^ until achieving convergence and determining their locations. The complete algorithm consists of two key components:

1. Sampling step.

(a) Set ${n}_{p}$ as the number of population size, ${n}_{s}$ as the number of sample size.

(b) In the space of ${[-90,90]}^{N}$, ${{n}}_{p}$ as $N$-dimensional vectors are uniformly generated, denote as $\Omega$.

(c) Choose an initial point *x*_*i*_ at random from $\Omega$.

(d) Choose the next point *x*_*i*_ with probability ($P\left({x}_{i}\right)=\frac{{D}^{2}({x}_{i})}{{\sum}_{x\in \Omega }{D}^{2}(x)} ,$ where $D({x}_{i})$ is the minimal distance from *x*_*i*_ to all sampled points).

(e) Repeat (d) until *n*_*s*_ points have been chosen in total.

2. Evaluation step.

(a) For *i* = 1… *n*_*s*_, Do,

(b) Let *x*_*i*_ be the starting point,

(c) *x*_*i*_ = *LS* (*x*_*i*_), where *LS*() is a local search procedure.

(d) End For.

The algorithm’s convergence is guaranteed: as initial points increase, the probability of finding the global optimum approaches 1. With p as the probability of a single point converging to the global minimum, ${n}_{s}$ points yield a success probability of ${1-(1-{{{\rm{p}}}})}^{{n}_{s}}$, approaching 1 as ${n}_{s}$ increases. This foundation, shown in Supplementary Information S2.3, ensures reliable identification of the global minimum NPR, and the convergence efficiency is demonstrated in section S2.1 and Table S2 in the Supplementary Information.

Following this, the minimum values obtained from all iterations are sorted to identify the minimum value of PR. For each $N$, the top 10 layups with lowest PR are outputted, serving as high-quality data for subsequent feature selection and clustering.

### Clustering

The feature identification follows Occam’s Razor Principle^40^, which advocates for simplicity and elegance in optimal scientific solutions. To align with engineering preferences, several indicator variables (valued as 0 or 1) are selected for layup and angles (please see Fig. 3b): (a) if the sum of angles equals 0, (b) if the layup is symmetric, and (c) if the layup is asymmetric and so on. Additionally, a quantitative variable representing the minimum NPR is set as our research object. An interesting finding from initial research is that different angles do not always increase with larger $N$; some angles only differ in sign, meaning their absolute values are the same. Consequently, we define another quantitative variable to capture the number of various angles in the following research.

Finally, clustering in ML is used to further analyze the features of layups associated with the minimum NPR. Hierarchical clustering^41^ is preferred over k-means clustering because it can reveal hierarchical relationships and multi-scale features through dendrogram visualization (Fig. 3b), which k-means cannot provide. Clustering is performed for $N=3$ to $20$, as the optimal solution for the PR is not negative when $N=2$. Detailed steps of hierarchical clustering algorithms are provided in Supplementary Information S2.2, with data presented in Table S3 to comprehensively validate these clustering results and their physical significance.

### High-dimension optimization

For higher-dimensional spaces (Fig. 3c), the mathematical principles derived from lower-dimensional spaces can be used as constraints during resampling to further optimize the algorithm. This approach can improve the efficiency of convergence by reducing the search space and guiding the algorithm toward more promising regions. By incorporating these lower-dimensional insights, the resampling process becomes more focused and effective, leading to quicker identification of optimal solutions. For the category of $N=4K$（*K* is a positive integer）, the layup for achieving the minimum NPR can be simplified to a pattern of $[-\theta /\theta /\theta /-\theta ]_{K}$. This reduction means that the number of variables decreases from $4K$ to just 1. Consequently, the search space contracts from ${\left[-{90}^{\circ },{90}^{\circ }\right]}^{4K}$ to $\left[-{90}^{\circ },{90}^{\circ }\right]$. This simplification not only reduces computational complexity but also enhances the efficiency of finding the optimal solution by focusing on a narrower range of angles. Through the comparison on the experiment with $N=100$, the optimized algorithm demonstrated efficiency improvements, reducing the search time to just $1/30$ of that required by the original algorithm. This inverse-design-based reduction in computation time underscores the effectiveness of optimization process. Similar in the category of $N=2K+1$, the layup is symmetric but number of independent angles is 2. Hence, the number of variables decreases from $2K+1$ to just 2. Consequently, the search space contracts from ${\left[-{90}^{\circ },{90}^{\circ }\right]}^{2K+1}$ to ${\left[-{90}^{\circ },{90}^{\circ }\right]}^{2}$. Through the comparison on the experiment with $N=99$, the optimized algorithm demonstrated efficiency improvements, reducing the search time to rougly $1/55$ of that required by the original algorithm. For the categories of $N=4K+2$, the number of variables is reduced only by half since only antisymmetric layups can be used to optimize the algorithm. Correspondingly, the search space contracts from ${\left[-{90}^{\circ },{90}^{\circ }\right]}^{4K+2}$ to ${\left[-{90}^{\circ },{90}^{\circ }\right]}^{2K+1}$. After comparison, the calculation efficiency is only improved by a factor of two. This indicates that while there is still a reduction in computational effort, the improvement is less dramatic than in the $N=4K$ categories due to the lesser reduction in dimensionality.

### Fabrication of LCSs

The LCS specimens used in this study are fabricated by placing each angled IM7/977-3 carbon fiber prepreg layup between upper and lower caul plates, followed by curing in an autoclave (Mini-Bonder 36 HP, USA) according to the manufacturer’s recommended cycle. The cycle involved increasing the temperature to 177 °C at a heating rate of 2.8 °C/min, holding it at 177 °C for 6 hours, and then cooling at 2.8 °C/min. The angled layups corresponded to the three identified families, $2K+1$,$\,4K$, and $4K+2$, at varying number of layers. The average ply thickness is 0.08 mm. The IM7/977-3 carbon fiber/epoxy prepregs exhibit an average fiber volume fraction ranging from 56% to 61%, as determined by preliminary optical microscopy imaging. The void content is ~2%. These values are consistent with those reported in the literature for composites fabricated using the same prepreg and autoclave curing technique^42,43^. The fiber volume fraction and void content influence the ply-level engineering constants (*E*_1_, *E*_2_, *v*_12_, *v*_21_, and *G*_12_), which are validated in this study by comparing the effective Poisson’s ratio predicted by Classical Laminate Theory and FEA with experimental results (see Results and Discussion section “Verification of inverse design”). After curing, the specimens are demolded and trimmed to a size of 3 mm by 3 mm.

### Finite element analysis

**Fig. 4:**
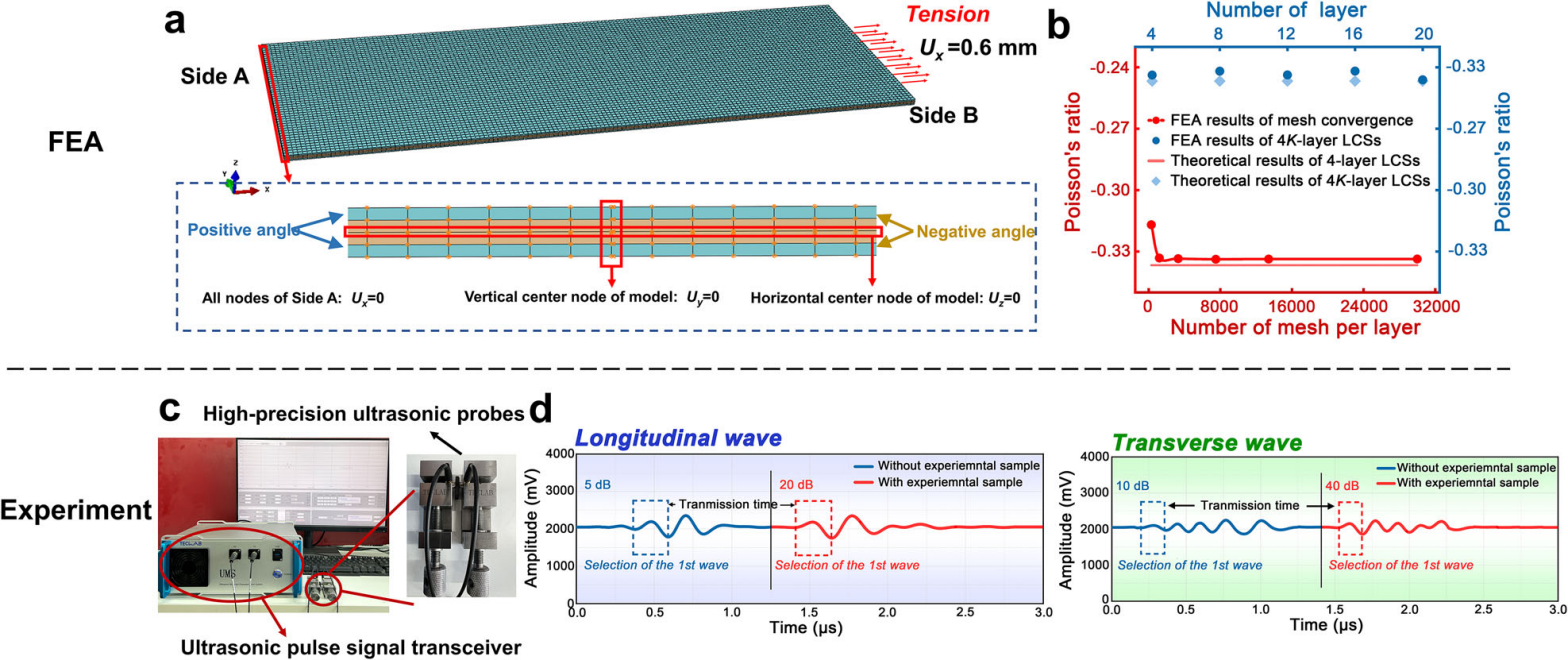
Finite element model and ultrasonic testing setup. **a** Boundary conditions and load of the finite element model for 4*K*-layer LCSs. **b** Mesh convergence results of Finite element analysis (FEA) (red horizontal and vertical axes) and the comparison between the theoretical results of 4*K*-layer LCSs and the FEA results (blue horizontal and vertical axes). **c** Experimental apparatus for performing non-destructive ultrasonic testing. **d** Schematic diagrams of longitudinal and transverse wave measurements of the speed of sound.

FEA is performed using commercial software Abaqus to illustrate strain-driven mechanism. Due to the most remarkable auxetic behavior, the model of the 4-layer three-dimensional LCS [24.5/-24.5/-24.5/24.5] is shown in Fig. 4a, where all elements are chosen to be of type C3D8R, with a length of 60 mm in the X-direction, a width of 20 mm in the Y-direction, and a thickness of 0.08 mm per layer. The material properties are set identically to those of IM7/977-3. To determine the PR of the LCS, a simulation of $1 \%$ tensile strain in the X-direction is performed. To ensure the model extends along the length direction, all nodes on one side (side A) are restricted in the *X*-direction $\left({U}_{x}=0\right)$, while a displacement load of 0.6 mm is applied on the opposite side (side B). Additionally, the central nodes in the vertical array on side A are constrained in the Y-direction (${U}_{y}$ $=0$), and all central nodes in the horizontal array are restricted in the Z-direction $\left({U}_{z}=0\right)$ to allow the model to deform smoothly.

Additionally, mesh convergence analysis is also performed. The results, depicted in Fig. 4b, compare the simulated data with theoretical results, demonstrating that stability and accuracy are ensured when the number of mesh elements per layer is at least 4800. Consequently, hexahedral meshes with 7500 elements per layer are employed for further analyses of the LCSs. The FEA results for all $4K$-layer LCSs within 20 layers, compared with GHL theoretical outcomes in Fig. 4b, show correlation, effectively verifying the precision of the ML results.

### Non-destructive ultrasound detection

The PR of LCSs in the out-of-plane direction is measured by using ultrasound, as shown in Fig. 4c. The experiment utilizes longitudinal and transverse ultrasonic pulses propagating through the LCS samples to measure their velocities. LCS samples with uniform thickness and flat surfaces are selected, ensuring consistency in the measurement direction. High-precision ultrasonic transducers (UMS−100, Ultrasonic Material Characterization System, China) are used to measure the propagation times of the longitudinal $\left({V}_{l}\right)$ and transverse $\left({V}_{t}\right)$ waves, as illustrated in Fig. 4d. After conducting frequency and velocity stability validation, based on the propagation times and sample thickness, the velocities of the longitudinal and transverse waves are calculated. Finally, the out-of-plane PR can be calculated using the measured wave velocity data

5 $$\nu =\frac{{V}_{l}^{2}-2{V}_{t}^{2}}{2\left({V}_{l}^{2}-{V}_{t}^{2}\right)}.$$

This experiment is carried out for several repeated measurements. The difference in the results of each measurement is within the allowable error, showing good repeatability and stability. The specific results are shown in Supplementary Information, Table S5, and the insensitivity of wave velocity to ultrasonic frequency is demonstrated in Supplementary Information S4 and Table S4.

### Digital image correlation tests

The digital image correlation (DIC) method is employed to measure both normal and shear strains on the sample’s surface by tracking the displacement of a speckle pattern. In this study, a uniform black-on-white speckle pattern is applied to the surface of the sample, which has an effective working area of 25 mm by 25 mm. The speckle pattern is optimized to achieve individual speckle sizes of 3-5 pixels in diameter, with approximately 40% black coverage to ensure optimal contrast. Reinforcement tabs are attached to both ends of the sample to ensure a uniform distribution of tensile force. A tensile strain is applied during the test.

The DIC system (XTDIC-CONST-HR, China) captured high-resolution images of the speckle pattern before and during deformation at a frame rate of 5 Hz using a camera with 35 mm focal length lens. These images are processed to calculate displacement fields and, subsequently, strain fields, including both tensile and shear strains. The DIC analysis parameters are carefully selected based on preliminary optimization: a subset size of 31 × 31 pixels (0.8 × 0.8 mm), step size of 7 pixels, and a strain calculation window of 5 data points.

Quality assessment showed excellent correlation throughout the tests, with correlation coefficients ranging from 0.94 to 0.99 (average: 0.97). The system achieved a displacement measurement accuracy of 0.05 pixels. The analysis focuses on the deformation across the effective area, allowing for accurate assessment of both normal and shear strain distributions under the applied load.

## Results and discussion

### Three LCS categories on minimum NPRs

**Fig. 5:**
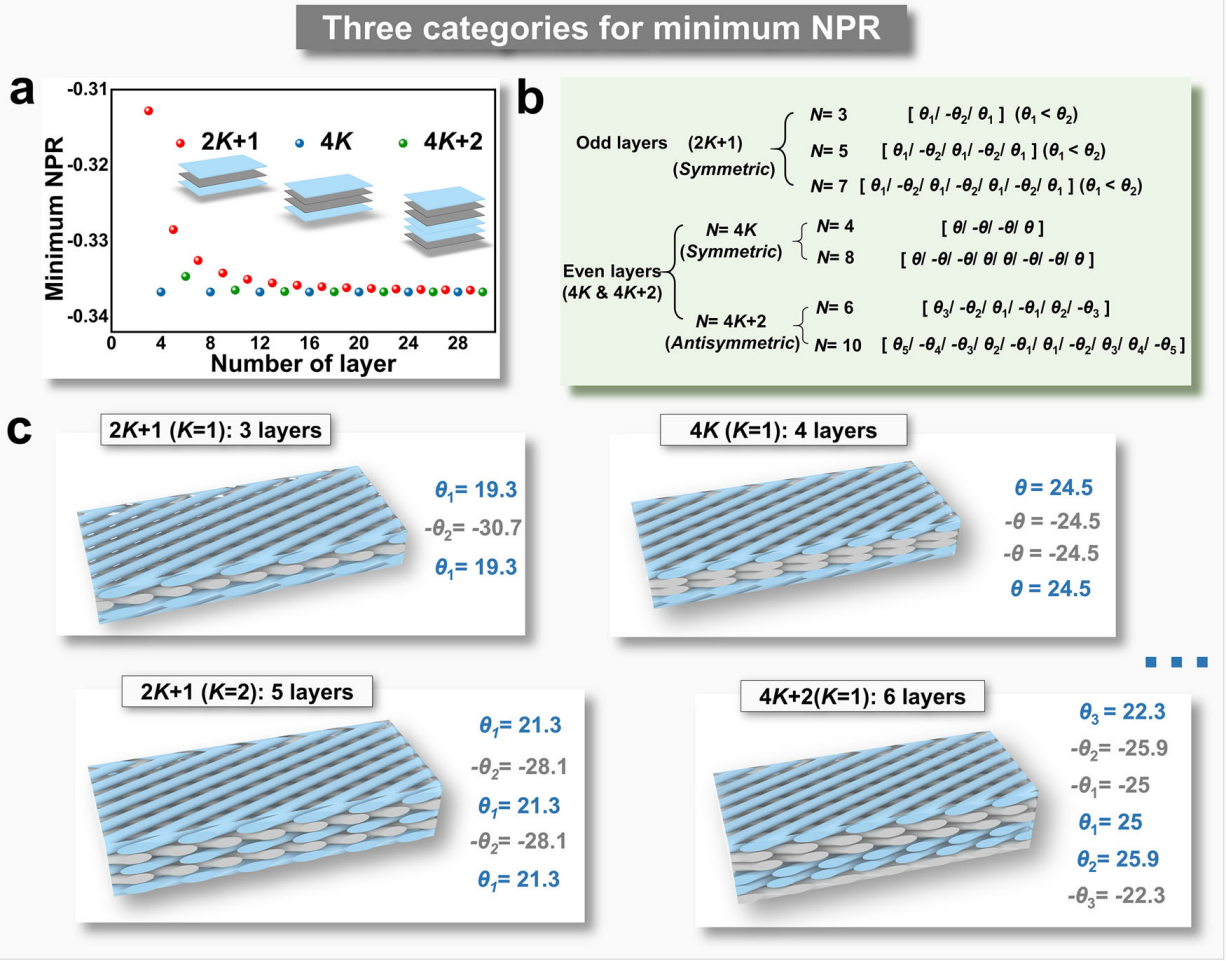
Layer number categories and layup characteristics based on minimum negative Poisson’s ratios (NPRs). **a** Three categories of layer numbers classified based on the relationship between minimum NPRs and the number of layers. **b** A summary of the layup characteristics for these three categories. **c** Representative layups for the three categories.

Figure 5a provides a graphical representation, offering a clear insight into the minimum NPR values across LCSs with varying layer counts. Based on the results of the multi-start resampling algorithm and clustering analysis, the LCSs can be distinctly categorized into three groups according to the number of layers and their corresponding minimum NPR values.

As shown in Fig. 5a, all LCSs with layer counts divisible by 4$K$ is a positive integer exhibit a minimal NPR of −0.3367. Furthermore, within this family, all layups achieving the minimal NPR are symmetric, with lamination angles of equal absolute value, as depicted in Fig. 5b, c. The second category, comprising odd-layer LCSs (denoted as $2K+1$-layer LCS), demonstrates the highest curve in the relationship between minimal NPR and the number of layers. Clustering analysis reveals that the layups in this category are also symmetric, with the axis of symmetry located at the center of the middle layer. These layups contain lamination angles with only two distinct values: one positive and one negative, with positive angles consistently fewer than negative ones by one. The final category, $4K+2$-layer LCSs, displays a similar but slower decline trend in minimum NPR compared to the $2K+1$-layer LCSs. In terms of angle combinations, these configurations follow an antisymmetric pattern, where each pair of antisymmetric angles has distinct absolute values. The layup structure for the $4K+2$-layer LCS is characterized by strict antisymmetry.

The classification of the three LCS categories based on minimum NPRs is derived from observations in Fig. 5a and summarized in Fig. 5b (The corresponding layups can be found in Supplementary Information, Table S1). In practical design implementation, maximum auxetic effects can be obtained by following these layup parity design principles. However, it is important to recognize that the main contribution to this discovery comes from the ML-aided inverse design method, which helps us uncover the physical rules hidden within complex mathematics. Furthermore, the proposed inverse design presents an efficient way for optimizing other physical properties of similar LCS structures. The 4*K*-layer family is found to achieve the extreme auxetic characteristics for LCSs with the simplest layup, which made it possible to solve the lamination angle in pure mathematics to verify the accuracy of inverse design results. This mathematical verification process is detailed in Supplementary Information, Discussion S3. Additionally, the reliability of the inverse design principles is further supported by statistical validation of the three family classifications through confidence level analysis, as presented in Supplementary Information S2.4, Tables S6 and S7.

### Verification of inverse design

To evaluate the effectiveness of the results derived from ML, we employed theoretical calculations, finite element analysis (FEA), and experimental methods to validate the accuracy of the results obtained in the last section from different perspectives. LCS samples from three categories are fabricated and characterized using the ultrasonic detection method (UDM)^44^.

**Fig. 6:**
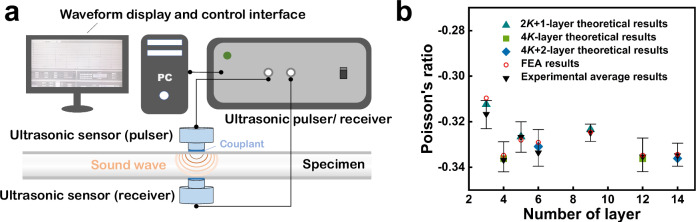
UDM experiment schematic and comparison of experimental measurements with Finite element analysis (FEA) and generalized Hooke’s law (GHL). **a** Schematic diagram of the ultrasonic detection method (UMD) experiment. **b** The comparison of the UDM experimental measurements, FEA results and the GHL numerical results. Error bars represent ± one standard deviation calculated from 4 independent experiments.

Considering the manufacturing process, lamination angles of LCS specimens for UDM are adjusted in 5° increments to reflect typical manufacturing tolerances and practical engineering constraints^45^. At least two representative layups from the categories $2K+1,\,4K$, and $4K+2$ are selected to ensure a minimum of two reference data points per category. At the same time, four replicates are used for each category to account for statistical variations. The PR values of the LCSs are tested using the non-destructive UDM method (Fig. 6a), which are calibrated by measuring the velocities of propagation of longitudinal and transverse waves inside the specimens. After performing mesh convergence and model accuracy of FEA, the comparison between theoretical, FEA and experimental results is shown in Fig. 6b. The outcomes reveal strong similarities and consistent trends, successfully validating the reliability of the ML results from both FEA and experimental perspectives.

In summary, our integrated approach combining simulation and experimental methods robustly validates the ML-derived NPR layup patterns. These findings not only confirm the accuracy of our ML models but also illustrate their potential in addressing complex numerical challenges and advancing future engineering designs.

### Auxetic mechanism of LCSs

The accuracy of angle combinations yielding minimum NPR in LCSs has been established, but understanding the overall auxetic deformation of LCSs at specific lamination angles remains a subject of exploration. This chapter investigates the deformation mechanisms of NPR in LCSs using FEA and Digital Image Correlation (DIC)^46,47^.

**Fig. 7:**
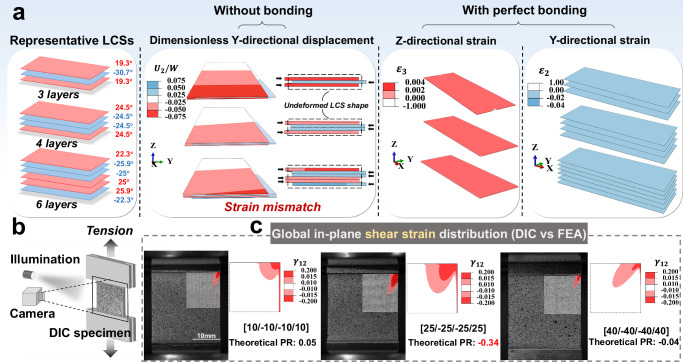
Finite element analysis (FEA) and Digital Image Correlation (DIC) of auxetic behavior in layered composite structures (LCSs): Deformation mechanisms and shear strain correlation. **a** The FEA results of deformation mechanism of LCSs exhibiting negative Poisson’s ratio (NPR). In the absence of bonding, three representative LCSs demonstrated layer separation and strain mismatch. With perfect bonding, the strain in the Z-direction is positive, while the strains in the Y-direction for all layers are negative. To reveal the relationship between the minimum NPR and strain. **b** Schematic diagram of the DIC experiments. **c** Comparison of shear strain cloud map between DIC and FEA results for a quarter range on the top right of the specimen, indicating the correlation between NPR and in-plane shear strain.

To elucidate why LCSs exhibit global auxetic behaviors, FEA is performed on three representative LCSs configurations, as illustrated in Fig. 7a. Specifically, the LCSs ($K$= 1) with layup [19.3/−30.7/19.3], [24.5/−24.5/−24.5/24.5], and [22.3/−25.9/−25/25/25.9/22.3] are analyzed using the same model settings as described in the preceding section. Each of these configurations is selected to represent varying degrees of ply orientation and symmetry, which are critical in influencing auxetic responses under mechanical loading. In Fig. 7a, assuming no bonding, all layers in each LCS experience lateral displacement due to shear deformation caused by tension. This response behavior to tension is quantitatively demonstrated through the dimensionless Y-direction displacement (Y-direction displacement divided by the width of the LCS), effectively illustrating the phenomenon of strain mismatch among the layers. Typically, bonding in LCSs is assumed to be perfect until damage occurs. Consequently, with bonding present, compressive forces arise in the Y-direction to counteract strain mismatches, leading to overall Z-directional expansion with auxetic effects manifesting globally throughout the LCSs. Under these conditions, all three types of representative LCSs exhibit auxetic deformation, characterized by positive strains in the Z-direction and negative strains in the Y-direction across all layers. The connection between tensile-shear deformation, strain mismatch, and compressive strain underscores a fundamental strain-driven mechanism that is the origin of the auxetic behavior observed in these LCSs.

Additionally, to elucidate the relationship between shear strain and auxetic performance, a comparative analysis is conducted using DIC (Fig. 7b) experiments and FEA. For the convenience of specimen preparation and outcome variance, tensile tests are carried out on three $4K$-layer LCSs with layups of [10/−10/−10/10], [25/−25/−25/25] and [40/−40/−40/40]. Theoretically predicted PRs for these three LCSs are 0.05, −0.34 and −0.04, respectively. As illustrated in Fig. 7c, DIC (three specimens per group shown in Supplementary information Fig. S1) is employed to map the shear strain distribution on the laminate surface, and the comparison between DIC and FEA shear strain contours demonstrated strong agreement in the in-plane shear strain distribution, notably with elevated concentrations at the edges. The most valuable finding is the correlation between NPR and shear strain, where greater shear strain corresponds to more pronounced auxeticity. Since all layers use identical IM7/977-3 material, modulus mismatch is not a dominant factor in the system. Sensitivity analysis (as shown in the Supplementary Information Fig. S2, Table. S8-S10) confirms that ply orientation mismatch alone drives the auxetic behavior, with angular configuration showing dominant effects while material properties have negligible influence, validating our strain-driven mechanism. In-plane shear strains magnitude responds to layer-to-layer strain mismatch, therefore, this strain-driven mechanism of auxetic deformation is once again confirmed.

In summary, through the application of DIC and FEA, the deformation mechanism of LCSs exhibiting NPR has been successfully elucidated. The strain-driven NPR mechanism revealed in this study is distinct from that of other man-made auxetic metamaterials^10,48–53^, whose auxetic behaviors are primarily attributed to engineered geometric topological lattices. As a result, auxetic materials can achieve NPR through a strain-driven mechanism without sacrificing material properties. This strain-driven NPR mechanism not only provides a perfect explanation for the auxetic behaviors of biomimetic LCSs but also opens up inverse-design-based avenues for the design and application of auxetically architectured structures.

## Supplementary information

Supplementary Information

## Data Availability

The detailed data sets are available at GitHub (https://github.com/lrwlx/minimum_NPRs).

## Code availability

The source code is available at GitHub (https://github.com/lrwlx/minimum_NPRs).
