# Supplementary material for: Machine learning-enabled inverse design of bioinspired layered composite structures with maximum auxetic performance
Source: Commun Eng. 2025 Nov 24;4:223. doi: 10.1038/s44172-025-00557-5 (PMC12749531; doi:10.1038/s44172-025-00557-5)
Supplement: Supplementary file 1 — Supplementary Information [file 44172_2025_557_MOESM1_ESM.pdf]

Supplementary Information

Machine learning-enabled inverse design of bioinspired layered composite structures with maximum auxetic performance

Yuze Li[1†], Rui Li[2†], Yin Fan[1*], Zhouyu Zheng[1], Hui-Shen Shen[1],

Xiuhua Chen[1], Minhua Wen[2], James Lin[2], Woong-Ryeol Yu[3]

and Yeqing Wang[4]

[1*] School of Aeronautics and Astronautics, Shanghai Jiao Tong

University, Shanghai 200240, China.

[2] High Performance Computing Center, Shanghai Jiao Tong University,

Shanghai 200240, China.

[3] Department of Materials Science and Engineering, Research Institute of

Advanced Materials, Seoul National University, Seoul 08826, Rep of Korea.

[4] Department of Mechanical and Aerospace Engineering, Syracuse

University, Syracuse, NY 13244, USA.

*Corresponding author. E-mail: yfan1987@sjtu.edu.cn;

[†] These authors contributed equally to this work.

This PDF file includes:

- Supplementary Methods
- Fig. S1 to S2
- Tables S1 to S10
- References (1 to 4)

Supplementary Methods

S1 Poisson's ratio for layered composite structures calculation based on classical laminate theory

In this paper, the IM7/977-3 [1-3] was selected as the prepreg for layered composite structures (LCSs). The specific material parameters used for both theoretical and numerical analysis are shown in Table S1.

The findings concerning the minimum negative Poisson's ratios (NPR) and the lamination angles of low-dimensional layered composite structures (comprising up to 10 layers) determined through the multi-start resampling algorithm are presented in Table S1.

We concentrate on Poisson's ratio (PR) ν_{13}, which is significantly affected by stiffness. According to generalized Hooke's law and the classical lamination theory (CLT), the out-of-plane PR can be calculated by the following equation [4]

$$\nu_{13} = -\frac{J_{31}}{J_{11}}, \tag{S1}$$

where J_{ij} are elements in the (i, j) entry of matrix $\mathbf{J}$. The exact process will be derived and written in brief below.

For an LCS with arbitrary layup, the relation between load and deformation are written as

$$\begin{Bmatrix} \mathbf{N} \\ \mathbf{M} \end{Bmatrix} = \begin{bmatrix} \mathbf{A} & \mathbf{B} \\ \mathbf{B} & \mathbf{D} \end{bmatrix} \begin{Bmatrix} \boldsymbol{\varepsilon} \\ \boldsymbol{\kappa} \end{Bmatrix}. \tag{S2}$$

From Eq. S2, we get

$$\begin{Bmatrix} \boldsymbol{\varepsilon} \\ \boldsymbol{\kappa} \end{Bmatrix} = \begin{bmatrix} \mathbf{A}^* - \mathbf{B}\mathbf{D}^{*-1}\mathbf{H}^* & \mathbf{B}\mathbf{D}^{*-1} \\ -\mathbf{D}^{*-1}\mathbf{H}^* & \mathbf{D}^{*-1} \end{bmatrix} \begin{Bmatrix} \mathbf{N} \\ \mathbf{M} \end{Bmatrix}. \tag{S3}$$

Without the exerted moment, the expression for the strain vector ε can be obtained as

$$\boldsymbol{\varepsilon} = (\mathbf{A}^* - \mathbf{B}^*\mathbf{D}^{*-1}\mathbf{H}^*)\mathbf{N} = (\mathbf{A}^{-1} + \mathbf{A}^{-1}\mathbf{B}(\mathbf{D} - \mathbf{B}\mathbf{A}^{-1}\mathbf{B})^{-1}\mathbf{B}\mathbf{A}^{-1})\mathbf{N} = \mathbf{J}\mathbf{N}, \tag{S4}$$

where

$$\mathbf{J} = \mathbf{A}^{-1} + \mathbf{A}^{-1}\mathbf{B}(\mathbf{D} - \mathbf{B}\mathbf{A}^{-1}\mathbf{B})\mathbf{B}\mathbf{A}^{-1}. \quad (S5)$$

The elements of the aforementioned stiffness matrices $\mathbf{A}, \mathbf{B}$, and $\mathbf{D}$ can be defined as

$$\left(A_{ij}, B_{ij}, D_{ij}\right) = \sum_{k=1}^{N} \int_{h_{k-1}}^{h_k} \left(\bar{C}_{ij}\right)_k (1, Z, Z^2) dZ. \quad (S6)$$

In Eq. S6, $\left(\bar{C}_{ij}\right)_k$ represents the stiffness matrix of kth layer after angle transformation, which can be determined by the stiffness coefficients and the lamination angle θ.

The matrix $\overline{\mathbf{C}}$ (*1*) is symmetric, with all elements given by the following formula:

$$\begin{aligned}
\bar{C}_{11} &= C_{11}\cos^4\theta + (2C_{12} + 4C_{66})\cos^2\theta\sin^2\theta + C_{22}\sin^4\theta\,, \\
\bar{C}_{22} &= C_{22}\cos^4\theta + (2C_{12} + 4C_{66})\cos^2\theta\sin^2\theta + C_{11}\sin^4\theta\,, \\
\bar{C}_{33} &= C_{33}, \\
\bar{C}_{66} &= (C_{11} + C_{22} - 2C_{12} - 2C_{66})\cos^2\theta\sin^2\theta + C_{66}(\sin^4\theta + \cos^4\theta), \\
\bar{C}_{13} &= \bar{C}_{31} = C_{13}\cos^2\theta + C_{23}\sin^2\theta\,, \qquad (S7) \\
\bar{C}_{23} &= \bar{C}_{32} = C_{23}\cos^2\theta + C_{13}\sin^2\theta\,, \\
\bar{C}_{16} &= \bar{C}_{61} = (C_{11} - C_{12} - 2C_{66})\cos^3\theta\sin\theta + (C_{12} - C_{22} + 2C_{66})\cos\theta\sin^3\theta\,, \\
\bar{C}_{26} &= \bar{C}_{62} = (C_{11} - C_{12} - 2C_{66})\cos\theta\sin^3\theta + (C_{12} - C_{22} + 2C_{66})\cos^3\theta\sin\theta\,, \\
\bar{C}_{36} &= \bar{C}_{63} = (C_{13} - C_{23})\cos\theta\sin\theta\,.
\end{aligned}$$

S2 Convergence Proof for the Multi-start Resampling Algorithm

S2.1 K-means++ for Multi-start Resampling Algorithm convergence efficiency

The primary motivation for this choice was computational efficiency: k-means++ maintains computational complexity, in stark contrast to the complexity of Gaussian process adaptive sampling, which becomes computationally prohibitive for LCSs with more than 15-20 plies. Unlike simpler approaches such as random sampling or Latin hypercube methods, which produce uneven coverage with both clustered points and large blank regions in high-dimensional spaces, k-means++ uses a distance-weighted probability (is the minimum distance to previously selected points) to ensure more uniform exploration of the solution space. This approach directly addresses the trade-off between global vs local minima by systematically exploring the solution space while avoiding redundant local searches in the same basin of attraction. As shown in Table S2, this method consistently reduced function evaluations by 19.9-30.7% across five benchmark functions with known global minima (Rastrigin, Powell, Sinusoidal, Trid, and Rosenbrock), covering

problem dimensions ranging from 8 to 17. This improvement in efficiency directly translates to enhanced reliability in global optimization for LCS design problem.

S2.2 Hierarchical clustering algorithm

The clustering module utilizes the hierarchical clustering algorithm and includes the following steps.

Step 1: For layers $N = 3$ to 20, calculate three indicator variables and two numerical variables to characterize the angular distribution and the minimum NPR at its lowest point for each layer.

Step 2: Calculate the similarity between each layer based on features.

Step 3: Construct a clustering tree based on the distance matrix using the ward linkage criterion. The resulting hierarchy of clusters can be visualized as a dendrogram.

Step 4: Determination of the final clustering results by cutting the dendrogram at a certain height or distance threshold. The cutting height is chosen using the maximum breakpoint of all breakpoints selected by the silhouette coefficient.

As shown in Table S3, "if_ symmetric" metric contains fractional values (e.g., 0.8 for N=7, 0.6 for N=9) that precisely quantify the proportion of symmetric configurations among the top 10-performing layups. For N=7, this reflects that 80% of optimal layups were fully symmetric while 20% showed near-symmetric patterns like [22.2/-27/22.2/-27/22.2/-27/22.2] (from Table S1), which still achieve excellent NPR (-0.3326). Similarly, the representation of angle sums preserves near-zero values rather than enforcing strict binary classification. The odd-layered systems (2K+1 family) all show if_ zero _ sum _angles =0 not because they lack near-zero summation, but because they mathematically cannot achieve exact zero due to the central ply constraint.

The hierarchical clustering algorithm (Ward's method) naturally incorporates these transitional states through its distance-based metric, positioning partially symmetric configurations between fully symmetric and fully asymmetric clusters according to actual process quality and practical engineering requirements. This is evident in the fact that systems with partial symmetry (e.g., N=7, 8, 9, 11) consistently attain NPR values within 0.5% of the global optimal -0.3367, indicating that these nuanced correlations are preserved and actively contribute to auxetic performance rather than being obscured by our classification system.

S2.3 Convergence proof for the Multi-start Resampling Algorithm

The proof involves introducing two key concepts:

1. Convergence Probability p : This is a constant representing the probability that a randomly chosen initial point will eventually converge to the global minimum after iterations.

2. Successful Convergence Number K_c : This is a random variable representing the number of initial points that will eventually converge to the global optimal point.

Here we assume p is a constant and is strictly greater than zero, basing on the assumption that the function $f(x)$ is well-defined and has a global minimum.

When sampling n_s random initial points, the successful convergence number K_c follows a binomial distribution with parameters n_s and p, i.e., $K_c \sim \mathrm{Bin}(n_s, p)$. Then the probability of no successful convergence point is

$$P(K_c = 0) = (1 - p)^{n_s}. \qquad (S8)$$

Therefore, the probability of at least one initial point that converges to global minimum is

$$P(K_c > 0) = 1 - (1 - p)^{n_s}. \qquad (S9)$$

As n_s increases to infinity, $(1 - p)^{n_s}$ approaches 0. That is

$$\lim_{n_s \to \infty} P(K_c > 0) = 1. \qquad (S10)$$

This shows that with a large enough n_s, the probability that at least one initial point will converge to the global minimum approaches 1.

The proportion of successful convergences $\frac{K_c}{n_s}$ has a standard deviation (SD) given by

$$SD\left(\frac{K_c}{n_s}\right) = \sqrt{\frac{p(1 - p)}{n_s}}. \qquad (S11)$$

This indicates that as n_s increases, the standard deviation of the proportion of successful convergences approaches zero, reflecting diminishing variability.

S2.4 Statistical validation of the three family classifications

Statistical analysis of minimum NPR values across the three laminate families quantitatively validates the mechanical distinctiveness of the classification system. As summarized in Table S6,

the $4K$ family exhibits zero variance (σ=0.00000) across all layer counts (N=4,8,12,16,20), confirming that strict symmetry eliminates performance fluctuations and achieves perfect consistency at the theoretical optimum (-0.33673). In contrast, the $2K$+1 family demonstrates significantly higher variability (σ=0.00640), reflecting the influence of central-ply effects in odd-layered systems where NPR ranges from -0.31281 (N=3) to -0.33615 (N=19). The $4K$+2 family shows intermediate consistency (σ=0.00076) with performance monotonically improving from -0.33467 (N=6) to -0.33670 (N=18), indicating layer-dependent optimization potential.

Crucially, pairwise t-tests (Table S7) confirm all inter-family differences are statistically significant ($p<0.05$), with the largest performance gap between $4K$ and $2K$+1 families (mean Δ=0.00267, $p<0.001$). The non-overlapping 95% confidence intervals further demonstrate mechanical distinctiveness: $4K$ [-0.33673, -0.33673], $2K$+1 [-0.33849, -0.32963], and $4K$+2 [-0.33721, -0.33504]. These results substantiate that the classification captures fundamental differences in auxetic behavior: symmetry-driven invariance ($4K$), central-ply-induced variance ($2K$+1), and transitional layer-dependent optimization ($4K$+2).

S3 Analytical evidence for 4K LCSs

Highly regular results are typically achieved through numerical solutions; however, the accuracy of these results remains unvalidated, and the mechanisms underlying such regularity have not been fully elucidated. To tackle these critical issues, this section presents a detailed theoretical derivation of the minimum out-of-plane NPR for LCSs. The approach simplifies the task of locating the global minimum point to a polynomial root-finding problem. Remarkably, the optimal lamination angles deduced align precisely with those derived from the previously discussed numerical algorithm, providing indirect validation of the numerical algorithm's accuracy.

For the category $N = 4K$, there are four assumptions as follow:

1. LCS is symmetrically layered.

2. The thickness of each layer is consistent.

3. The absolute value of the angle of each layer is equal.

4. The number of layers with a positive angle equals the number of layers with a negative angle.

The symmetry in Assumption 1 leads to $\mathbf{B} = \mathbf{0}$, thereby simplifying the matrix $\mathbf{J}$ in the inverse matrix of $\mathbf{A}$. Then, the derivation of this numerical problem proceeds in two main steps. The first step is to use the assumptions to simplify the formula for calculating ν_{13} to a fraction that only relates to $\bar{C}_{ij}$. The second step is to utilize the properties of $\bar{C}_{ij}$ in terms of sine and cosine functions and apply the substitution method to transform the problem of finding the extremum of ν_{13} into a polynomial root-finding problem. The process is as follows:

The expression for each element of the matrix **A** is:

$$A_{ij} = \sum_{k=1}^{N} h\left(\bar{C}_{ij}\right)_k. \qquad (S12)$$

From Eq. S14, we can express it in matrix form as follows:

$$\mathbf{A} = h \sum_{k=1}^{N} \bar{\boldsymbol{C}}_k, \qquad (S13)$$

where **A** and $\bar{\boldsymbol{C}}_k$ are in matrix form which has been mentioned in Section 1 of SI. There exists an odd power term of $\sin(\theta)$, which lead $\bar{C}_{16}, \bar{C}_{26}$ and $\bar{C}_{36}$ to odd functions in matrix $\bar{\mathbf{C}}$. Therefore, the nature of the odd function of $\sin(\theta)$ can be utilized to transform $\bar{A}_{16}, \bar{A}_{26}$ and $\bar{A}_{36}$ to 0 under special conditions, which is a result of assumptions 3 and 4. This yields the **A** matrix as follows:

$$\mathbf{A} = 4Kh \begin{pmatrix} \bar{C}_{11} & \bar{C}_{12} & \bar{C}_{13} & 0 \\ \bar{C}_{21} & \bar{C}_{22} & \bar{C}_{23} & 0 \\ \bar{C}_{31} & \bar{C}_{32} & \bar{C}_{33} & 0 \\ 0 & 0 & 0 & \bar{C}_{66} \end{pmatrix}. \qquad (S14)$$

With the above conditions satisfied, the values of $\bar{A}_{16}, \bar{A}_{26}$ and $\bar{A}_{36}$ are all 0 and the elements of the **A** matrix are simplified. At the same time, **A** is symmetric matrix. Following, ν_{13} has the expression:

$$\nu_{13} = -\frac{J_{31}}{J_{11}} = -\frac{(\mathbf{A}^{-1})_{31}}{(\mathbf{A}^{-1})_{11}}. \qquad (S15)$$

According to the calculation method of matrix inverse, $\mathbf{A}^{-1}$ can be written as

$$\mathbf{A}^{-1} = \frac{1}{|\mathbf{A}|} \mathrm{adj}(\mathbf{A}), \qquad (S16)$$

where $|\mathbf{A}|$ is the determinant of **A**, $\mathrm{adj}(\mathbf{A})$ is the adjoint matrix of **A**. Thus,

$$J_{11} = \frac{\bar{C}_{66} \times (\bar{C}_{22}\bar{C}_{33} - \bar{C}_{23}\bar{C}_{32}) \times (4Kh)^3}{|\mathbf{A}|} \qquad (S17)$$

$$J_{31} = \frac{\bar{C}_{66} \times (\bar{C}_{12}\bar{C}_{23} - \bar{C}_{22}\bar{C}_{13}) \times (4Kh)^3}{|\mathbf{A}|} \qquad (S18)$$

Since $\bar{C}_{ij} = \bar{C}_{ji}$, the expression for ν_{13} can be simplified and written as:

$$\nu_{13} = -\frac{\bar{C}_{12}\bar{C}_{23} - \bar{C}_{22}\bar{C}_{13}}{\bar{C}_{22}\bar{C}_{33} - \bar{C}_{23}^2}. \qquad (S19)$$

In summary, we have finally simplified ν_{13} to an expression involving only $\bar{C}_{12}, \bar{C}_{13}, \bar{C}_{22}, \bar{C}_{23}$ and $\bar{C}_{33}$. Next, we will simplify $\bar{C}_{ij}$ as a function of θ.

In the expression of $\bar{\mathbf{C}}$, all $\bar{C}_{ij}$ has only two forms.

Form 1: $\bar{C}_{11}, \bar{C}_{12}, \bar{C}_{22}, \bar{C}_{66}$ can be transformed into a functional relation of the same form. i.e.,

$$f_1(\theta) = a\cos^4\theta + b\cos^2\theta\sin^2\theta + c\sin^4\theta\,, \qquad (S20)$$

where $a, b,$ and c are constants.

Form 2: $\bar{C}_{13}, \bar{C}_{23}$ can be transformed into another functional equation of the same form, i.e.,

$$f_2(\theta) = p\cos^2\theta + q\sin^2\theta\,, \qquad (S21)$$

where $p$and q are constants.

The first order derivatives of forms 1 and 2 can be expressed as:

$$f_1'(\theta) = [2(a - b + c)\sin^2(\theta) + (b - 2a)]\sin(2\theta)\,, \qquad (S22)$$

$$f_2'(\theta) = (q - p)\sin(2\theta)\,. \qquad (S23)$$

Denoting $\sin^2(\theta) = x$, we can transform f_1, f_2, and their derivatives into:

$$f_1 = (a - b + c)x^2 + (b - 2a)x + a, \quad (S24)$$
$$f_1' = [2(a - b + c)x + (b - 2a)]\sin(2\theta), \quad (S25)$$
$$f_2 = (q - p)x + p, \quad (S26)$$
$$f_2' = (q - p)\sin(2\theta). \quad (S27)$$

Next, we can transform the issue of solving the minimum value of ν_{13} into a matter of calculating the extreme value of ν_{13}. Using the quotient rule for differentiation, the derivation of ν_{13} can be written as

$$(\nu_{13})' = -\frac{((\bar{C}_{12}\bar{C}_{23} - \bar{C}_{22}\bar{C}_{13})'(\bar{C}_{22}\bar{C}_{33} - \bar{C}_{23}^2) - (\bar{C}_{12}\bar{C}_{23} - \bar{C}_{22}\bar{C}_{13})(\bar{C}_{22}\bar{C}_{33} - \bar{C}_{23}^2)')}{(\bar{C}_{22}\bar{C}_{33} - \bar{C}_{23}^2)^2}. \quad (S28)$$

Let

$$h(\theta) = \bar{C}_{12}\bar{C}_{23} - \bar{C}_{22}\bar{C}_{13}, \quad (S29)$$
$$g(\theta) = \bar{C}_{22}\bar{C}_{33} - \bar{C}_{23}^2. \quad (S30)$$

When $(\nu_{13})' = 0$, ν_{13} has critical points that may be extrema. Therefore, the points where derivatives are zero can be solved to find the corresponding θ values. Consequently, we denote the numerator of $(\nu_{13})'$ as $F(\theta)$, then

$$F(\theta) = h'(\theta)g(\theta) - h(\theta)g'(\theta), \quad (S31)$$

where both terms of $h'(\theta)$ and $g'(\theta)$ contain $\sin(2\theta)$. Thus $F(\theta)$ can be extracted as a common factor in the expression for $\sin(2\theta)$, transforming the problem entirely into a problem of solving the zeros of a fourth-degree polynomial in x, where $x = \sin^2(\theta)$. Following, it is sufficient to solve for the value of θ at $(\nu_{13})' = 0$.

After solving this polynomial equation, which has a pair of conjugate complex roots, a negative root, and only one valid solution, 0.1687, falls in the interval [0,1]. Solving again for $\sin^2(\theta) = 0.1687$, the final result solves with $\theta = 24.5°$ and $-24.5°$.

S4 Insensitivity of wave velocity to ultrasonic frequency

Systematic pre-experiments were performed on samples of different materials using a range of transducer frequencies to comprehensively evaluate potential frequency dependence as shown in Table S4. Specifically, three representative materials were tested across a frequency range of 1-

10 MHz: Sample No.1 (glass), Sample No.2 (stainless steel 304), and Sample No.3 (multi-layer CFRP), with both longitudinal and shear wave velocities monitored.

The results demonstrated remarkable consistency: variations in transducer frequency had negligible effects on both longitudinal and shear wave velocity measurements. Regardless of material type, the calculated elastic moduli and Poisson's ratios remained highly stable throughout the tested frequency spectrum. These pre-measurement validations confirmed that frequency-dependent effects are negligible in the composite systems under investigation.

S5 Sensitivity analysis for dominant role of ply orientation mismatch in Strain-Driven Auxeticity

All LCS investigated in this study were fabricated from identical IM7/977-3 carbon fiber prepregs (Methods 2.2, Table 1), ensuring that all layers possess exactly the same material properties (moduli). Therefore, there is no modulus mismatch between layers in the LCS, eliminating it as a potential competing or dominant factor.

While the **J** matrix (Eq. S5: $\mathbf{J} = \mathbf{A}^{-1} + \mathbf{A}^{-1}\mathbf{B}(\mathbf{D} - \mathbf{B}\mathbf{A}^{-1}\mathbf{B})^{-1}\mathbf{B}\mathbf{A}^{-1}$) mathematically incorporates both material properties and ply orientations through the transformed stiffness coefficients $C_{ij}^{k}(p_k, a_k)$, in homogeneous material system, the complexity of the **J** matrix purely reflects how different ply orientations interact through the material's inherent anisotropy ($E_1/E_2 \approx 17.3$). The material properties remain constant across all layers, so the variation in $v_{13} = -J_{31}/J_{11}$ arises solely from the angular configuration of the plies. This is further evidenced by the transformed stiffness components such as $\bar{C}_{11} = C_{11}\cos^4\theta + (2C_{12} + 4C_{66})\cos^2\theta\sin^2\theta + C_{22}\sin^4\theta$, where the material constants (C_{11}, C_{12}, etc.) remain fixed while only the angular terms vary.

To quantitatively verify this mechanism, a Sobol sensitivity analysis was conducted with both ply angles and material properties (E_1, E_2, G_{12}, v_{12}, v_{13}) as input variables, incorporating ±50% uncertainty based on the original material properties (as shown in Tables S8-S10 and Figure S2). The results unequivocally demonstrate that ply angles exhibit dominant total effects (ST) ranging from 0.2 to 0.8, while material properties show negligible effects with ST values all below 0.05. The high total effects compared to first-order effects for ply angles indicate strong angular interactions, consistent with our strain-driven mechanism revealed through FEA and DIC experiments (Fig. 7).

These quantitative results confirm that in the homogeneous material system, ply orientation mismatch is the sole and dominant driver of strain redistribution leading to auxeticity.

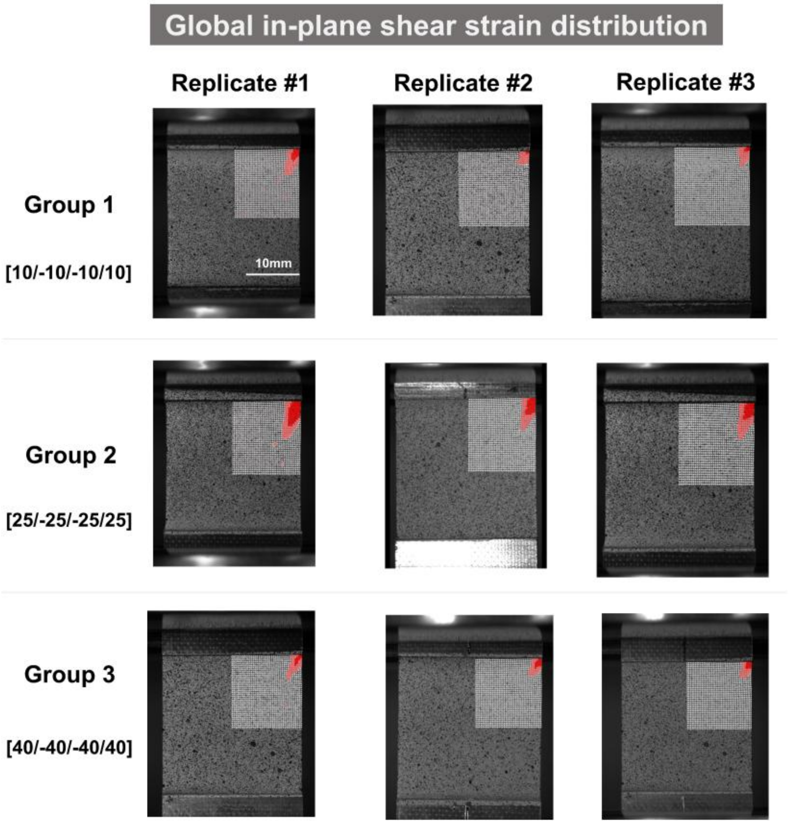

Fig S1. DIC shear strain contour maps for three groups of specimens (three specimens per group) in the upper right quadrant.

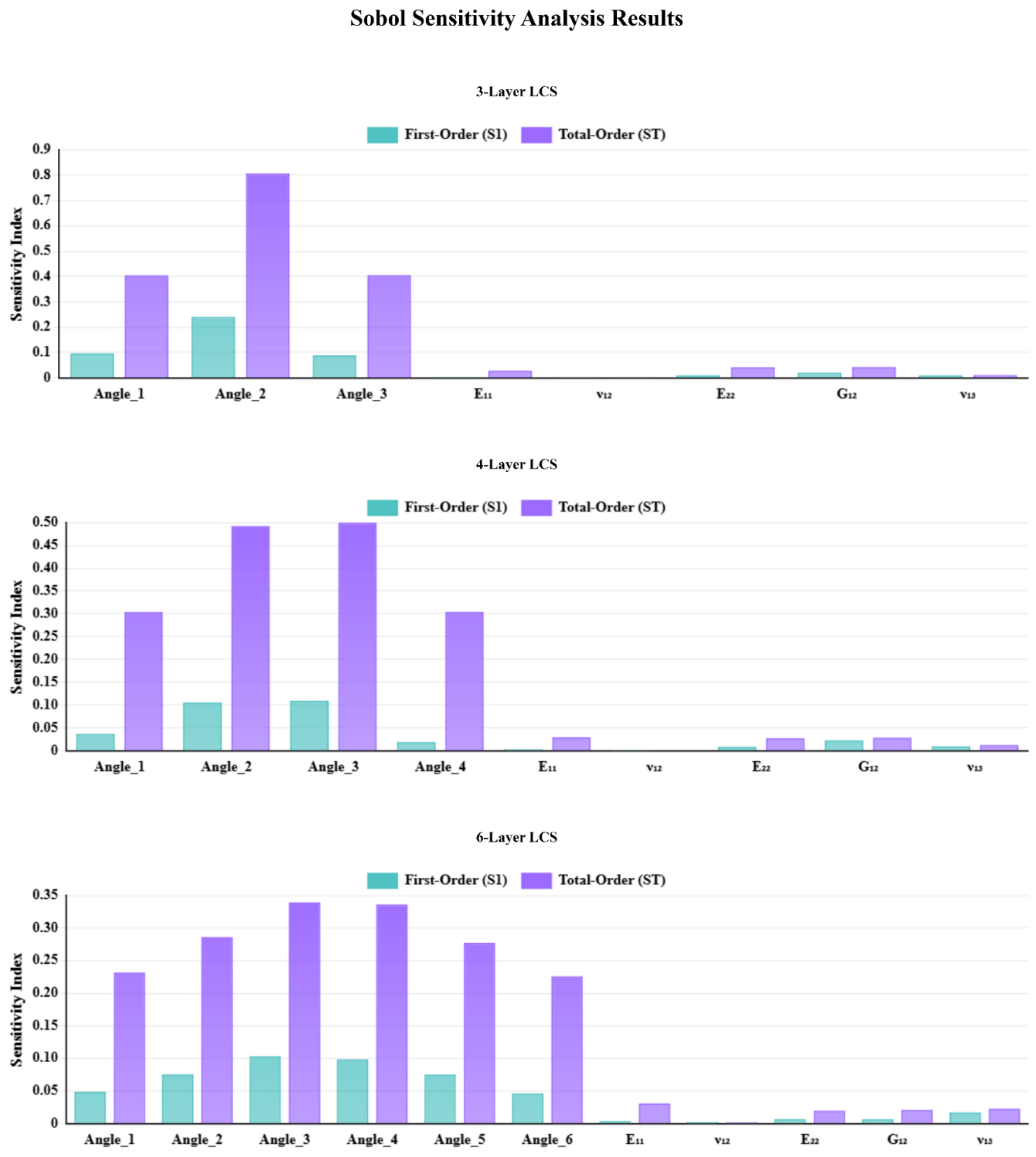

Fig. S2 Parameter sensitivity analysis for laminated composite structures with different layer configurations.

Table S1. Minimum NPRs and corresponding layups for LCSs with 3 layers to 10 layers

Number of layer	PR value	Layup
3(2*K*+1, *K*=1)	-0.3128	[19.3/-30.7/19.3]
4(4*K*, *K*=1)	-0.3367	[24.5/-24.5/-24.5/24.5]
5(2*K*+1, *K*=2)	-0.3285	[21.3/-28.1/21.3/-28.1/21.3]
6(4*K*+2, *K*=1)	-0.3347	[22.3/-25.9/-25/25/25.9/-22.3]
7(2*K*+1, *K*=3)	-0.3326	[22.2/-27/22.2/-27/22.2/-27/22.2]
8(4*K*, *K*=2)	-0.3367	[24.5/24.5/-24.5/-24.5/-24.5/-24.5/24.5/24.5]
9(2*K*+11, *K*=4)	-0.3342	[22.7/-26.5/22.7/-26.5/22.7/-26.5/22.7/-26.5/22.7]
10(4*K*+2, *K*=2)	-0.3365	[25.4/-23.8/-24/24.8/-24.4/24.4/-24.8/24/23.8/-25.4]
…	…	…

Table S2. Comparison of function call counts between Multi-Start (MS) and Multi-Start Resampling (MSRS) algorithms for high-dimensional optimization benchmarks.

Function	Dimension	MS Calls	MSRS Calls	Improvement (%)
Rastrigin	8	42319	33412	21.0
Powell	8	20552	14962	27.2
Sinusoidal	16	13	9	30.7
Trid	17	135024	108112	19.9
Rosenbrock	16	1385	1067	22.9

Table S3. Optimized feature attributes of bioinspired composite laminates across layer counts (*N*=2 to 20)

N	Min PR	If: sentence				Num different abs angles
		zero sum angles	symmetric	negative symmetric	abs symmetric	
2	0.05092	1	0	1	1	1
3	-0.31281	0	1	0	1	2
4	-0.33673	1	1	0	1	1
5	-0.32849	0	1	0	1	2
6	-0.33467	1	0	1	1	3
7	-0.33258	0	0.8	0	0.8	2
8	-0.33673	1	0.8	0.2	1	1
9	-0.33423	0	0.6	0	0.9	2
10	-0.33648	1	0	1	1	5.1
11	-0.33506	0	0.4	0	1	2
12	-0.33673	1	0.3	0	1	1
13	-0.33553	0	0.3	0	1	2
14	-0.33666	1	0	0.2	1	5.5
15	-0.33583	0	0.1	0	1	2
16	-0.33673	1	0.2	0	1	1
17	-0.33603	0	0.1	0	1	2
18	-0.33670	1	0	0	1	3.8
19	-0.33615	0	0	0	1	2.2
20	-0.33673	1	0	0	1	1.4

Table S4. Ultrasonic velocity and material properties at different probe frequencies

Sample No.	Frequency (MHz)	Longitudinal wave (m/s)	Transverse wave (m/s)	Poission's ratio
1 (Glass)	1	6865.45	4119.27	0.2187
	2.25	6865.45	4195.56	0.2019
	3.5	6865.45	4195.56	0.2019
	5	6865.45	4195.56	0.2019
	7.5	6865.45	4195.56	0.2019
	8	6865.45	4195.56	0.2019
	10	6865.45	4195.56	0.2019
2 (Stainless steel 304)	1	5757.01	3134.86	0.2892
	2.25	5757.01	3134.86	0.2892
	3.5	5730.23	3142.86	0.2848
	5	5757.01	3134.86	0.2892
	7.5	5757.01	3142.86	0.2877
	8	5757.01	3142.86	0.2877
	10	5757.01	3134.86	0.2892
3 (multi-layer CFRP)	1	2832.00	1388.13	0.3419
	2.25	2841.12	1390.14	0.3425
	3.5	2841.12	1390.24	0.3425
	5	2841.12	1390.24	0.3425
	7.5	2832.30	1392.37	0.3406
	8	2832.30	1394.50	0.3400
	10	2841.12	1392.37	0.3419

Table S5. Comparison of the experimental, numerical, and theoretical results for NPR

Sample No.	LCS family & Layup (°)	Long. Sound Vel. (m/s)	Trans. Sound Vel. (m/s)	ν_{13} Meas.	ν_{13} (Experimental)	ν_{13} (Numerical)	ν_{13} (Theoretical)
1-A	3 layers ($2K$+1) [20/-30/20]	2754.97	2166.67	-0.310673	-0.3166	-0.3096	-0.3124
1-B		2526.32	1990.78	-0.319167			
1-C		2628.57	2073.24	-0.323097			
1-D		2672.94	2103.70	-0.313809			
2-A	4 layers (4K) $[25/-25]_S$	2815.50	2228.22	-0.338084	-0.3368	-0.3347	-0.3362
2-B		2764.10	2175.28	-0.342201			
2-C		2714.55	2145.45	-0.328872			
2-D		2713.51	2147.59	-0.338270			
3-A	5 layers (2K+1) [20/-30/20/ -30/20]	2540.00	2001.97	-0.320040	-0.3267	-0.3281	-0.3266
3-B		2710.20	2141.97	-0.331947			
3-C		2839.74	2245.03	-0.333370			
3-D		2546.34	2007.69	-0.321603			
4-A	6 layers (4K+2) $[20/-25_2/25_2/$ $-20]$	2692.22	2130.81	-0.338414	-0.3335	-0.3291	-0.3309
4-B		2788.30	2207.41	-0.339535			
4-C		2814.46	2224.76	-0.332809			
4-D		2684.15	2117.24	-0.323433			
5-A	9 layers ($2K+1$) [20/-25/-25/ 20/20/20/ -25/-25/20]	2695.89	2127.57	-0.325626	-0.3249	-0.3246	-0.3234
5-B		2690.45	2122.61	-0.324271			
5-C		2535.06	2002.05	-0.328709			
5-D		2759.73	2175.66	-0.321043			
6-A	12 layers (4K) $[25/-25]_{3S}$	2807.55	2220.90	-0.336012	-0.3352	-0.3347	-0.3362
6-B		2737.11	2220.41	-0.335998			
6-C		2730.00	2162.38	-0.341884			
6-D		2598.79	2051.67	-0.327206			
7-A	14 layers (4K+2) $[-25/25_2/-25/25/$ $-25_2/25_2/-25/25/$ $-25_2/25]$	2652.85	2098.36	-0.335667	-0.3347	-0.3343	-0.3361
7-B		2714.89	2144.54	-0.329671			
7-C		2588.24	2046.51	-0.334055			
7-D		2480.38	1963.64	-0.339535			

Note: Subscript "s" denotes the symmetry.

Table S6. Descriptive Statistics of Minimum NPR by Family

Family	Sample Size	Mean NPR	Std. Deviation	95% CI
4*K*	5	-0.33673	0.00000	[-0.33673, -0.33673]
2*K*+1	9	-0.33406	0.00640	[-0.33849, -0.32963]
4*K*+2	4	-0.33613	0.00076	[-0.33721, -0.33504]

Table S7. Statistical Significance of Inter-Family Differences

Comparison	Mean Difference	t-value	p-value	Significance
4*K* vs 2*K*+1	0.00267	9.82	<0.001	Highly Significant
4*K* vs 4*K*+2	0.00060	3.15	0.012	Significant
2*K*+1 vs 4*K*+2	0.00207	6.74	<0.001	Highly Significant

Table S8. 3-layer LCS Sobol Sensitivity Analysis Results for v_{13} (with Material Properties)

Parameter	First-Order (S1)	Total-Order (ST)
Angle_1	0.0976	0.4050
Angle_2	0.2412	0.8069
Angle_3	0.0888	0.4057
E_1	0.0031	0.0288
v_{12}	0.0006	0.0006
E_2,	0.0104	0.0420
G_{12}	0.0211	0.0431
v_{13}	0.0093	0.0117

Table S9. 4-layer LCS Sobol Sensitivity Analysis Results for v_{13} (with Material Properties)

Parameter	First-Order (S1)	Total-Order (ST)
Angle_1	0.0371	0.3045
Angle_2	0.1055	0.4929
Angle_3	0.1097	0.4999
Angle_4	0.0190	0.3047
E_1	0.0036	0.0293
v_{12}	0.0001	0.0007
E_2,	0.0087	0.0278
G_{12}	0.0227	0.0287
v_{13}	0.0095	0.0126

Table S10. 6-layer LCS Sobol Sensitivity Analysis Results for v_{13} (with Material Properties)

Parameter	First-Order (S1)	Total-Order (ST)
Angle_1	0.0485	0.2319
Angle_2	0.0757	0.2863
Angle_3	0.1032	0.3396
Angle_4	0.0987	0.3363
Angle_5	0.0752	0.2772
Angle_6	0.0463	0.2260
E_1	0.0039	0.0311
v_{12}	0.0027	0.0019
E_2,	0.0069	0.0200
G_{12}	0.0067	0.0211
v_{13}	0.0172	0.0229

Supplementary references

[1] Li, X. et al. Assessment of failure criteria and damage evolution methods for composite laminates under low-velocity impact. *Compos. Struct.* **207,** 727-739 (2019).

[2] Mohammadi, B., Rohanifar, M., Salimi-Majd, D. & Farrokhabadi, A. Micromechanical prediction of damage due to transverse ply cracking under fatigue loading in composite laminates. *J. Reinf. Plast. Compos.* **36,** 377-395 (2017).

[3] Zhang, J. & Zhang, X. An efficient approach for predicting low-velocity impact force and damage in composite laminates. *Compos. Struct.* **130,** 85-94 (2015).

[4] Fan, Y. & Wang, Y. The effect of negative Poisson's ratio on the low-velocity impact response of an auxetic nanocomposite laminate beam. *Int. J. Mech. Mater. Des.* **17,** 153-169 (2021).
